# PAM3 protects against DSS-induced colitis by altering the M2:M1 ratio

**DOI:** 10.1038/s41598-020-63143-z

**Published:** 2020-04-08

**Authors:** Begum H. Horuluoglu, Neslihan Kayraklioglu, Debra Tross, Dennis Klinman

**Affiliations:** Cancer and Inflammation Program, National Cancer Institute, NIH, Frederick, MD 21720 USA

**Keywords:** Acute inflammation, Peritoneal macrophages

## Abstract

Inflammation of the gastrointestinal tract contributes to the development of inflammatory bowel disease (IBD). Human IBD is modeled by administering dextran sulfate sodium (DSS) to mice. In humans and mice, inflammatory M1 macrophages contribute to the progression of IBD whereas immunosuppressive M2 macrophages protect against colitis. The TLR2/1 agonist PAM3CSK4 (PAM3) induces human and murine monocytes to differentiate into immunosuppressive M2 macrophages, suggesting that PAM3 might be of benefit in the prevention/treatment of colitis. PAM3 was therefore administered to mice treated with DSS. As hypothesized, the number of M2 macrophages rose and disease severity decreased. The critical role of M2 macrophages in this process was established by transferring purified M2 macrophages from PAM3 treated control donors into DSS recipients and reducing colitis. These findings suggest that PAM3 may represent a novel approach to the treatment of human IBD.

## Introduction

Ulcerative colitis and Crohn’s disease are chronic inflammatory disorders of the gastrointestinal tract^1^. In both types of IBD, activation of the innate rather than adaptive immune system is critical, with macrophages and dendritic cells contributing to the induction of inflammation^2–5^. Intestinal macrophages occupy the interface between the host’s GI tract and the resident microbiome. These macrophages can contribute to IBD pathogenesis by failing to eliminate inflammation-inducing microbes and/or failing to support the resolution of inflammation that arises via other mechanisms^6^. The DSS-induced model of murine colitis is widely used to study human IBD due to its rapidity, simplicity and reproducibility. DSS disrupts the colonic epithelium and facilitates the invasion of intestinal microbes through the mucosa, causing inflammation characterized clinically by weight loss, diarrhea and rectal bleeding^7–9^.

‘Classical’ or pro-inflammatory M1-like macrophages protect the host from infection whereas ‘alternatively activated’ M2-like macrophages act to suppress inflammation and support tissue remodeling^10–13^. Studies suggest that M1 and M2 macrophages have opposing roles in DSS-induced colitis^14^. M1 macrophages contribute to disease pathogenesis by secreting pro-inflammatory cytokines and causing tissue damage whereas M2 macrophages protect mice by secreting anti-inflammatory factors that aid in the resolution of inflammation^4,14–17^.

Depending upon the stimulus, monocytes can differentiate into either M1 or M2 macrophages. Our lab previously demonstrated that the TLR2/1 agonist PAM3CSK4 (PAM3) preferentially stimulated normal human and murine monocytes to mature into M2-like macrophages^10,18,19^. The murine M2 macrophages co-expressed CD206 and F480. Purified cells of this phenotype were functionally M2 (based on their production of IL-10, phagocytic activity, and inability to produce inflammatory cytokines)^10^. By comparison, F480^+^ macrophages that lacked CD206 were functionally M1 (producing IL-12 and IFNg, lysing tumor targets but lacking phagocytic activity)^18–22^. Further studies showed that weekly treatment with PAM3 delayed the progression of atuoimmune disease in (NZB x NZW) F1 mice by increasing the number of M2 versus M1 macrophages *in vivo*^10^. The current work was undertaken to investigate the effect of PAM3 on mice with DSS-induced colitis. Results show that the M2 macrophages generated by PAM3 treatment reduced disease severity in this model of IBD. As PAM3 has the same effect on primate as murine monocytes, these findings suggest that the PAM3-based therapy may be of use in the treatment of human colitis.

## Results

### PAM3 treatment reduces the severity of DSS induced colitis

**Figure 1:**
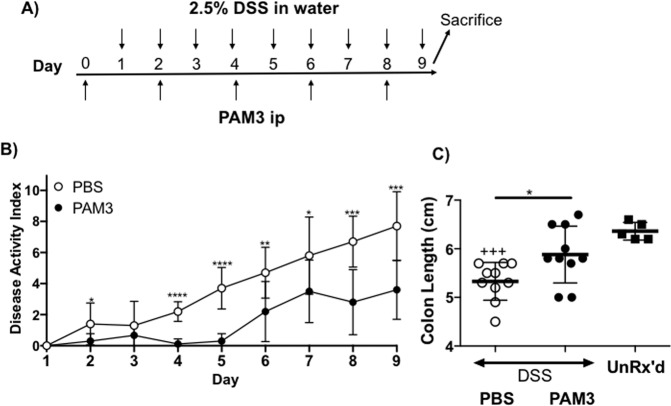
PAM3 treatment reduces disease severity. (**A**) C57/BL6 mice were treated with PAM3 (N = 10) or PBS (N = 10) every 2 days and with 2.5% DSS in drinking water daily starting on day 1 and continued through day 9 when they were sacrificed. (**B**) DAI was assessed daily. (C) Colon lengths were measured on day 9. Data represent the mean ± SD. Untreated controls were included for comparison (N = 5). *p < 0.05; **p < 0.01; ***p < 0.001 for PAM3 versus PBS treated groups. ^+++^p < 0.001 for PBS compared to untreated controls.

Colitis was induced by adding DSS to the drinking water of C57/Bl6 mice for 8 days. The level of inflammation this protocol elicited was determined daily using the “disease activity index” (DAI) and by measuring colon length at the end of treatment (inflammation causes significant shortening of the large intestine). As expected, mice given DSS developed extensive disease as measured by both criterion (Fig. 1). When animals given DSS were treated with PAM3, disease severity was significantly reduced and colon length remained normal (Fig. 1).

### Effect of PAM3 on M2 macrophage frequency

**Figure 2:**
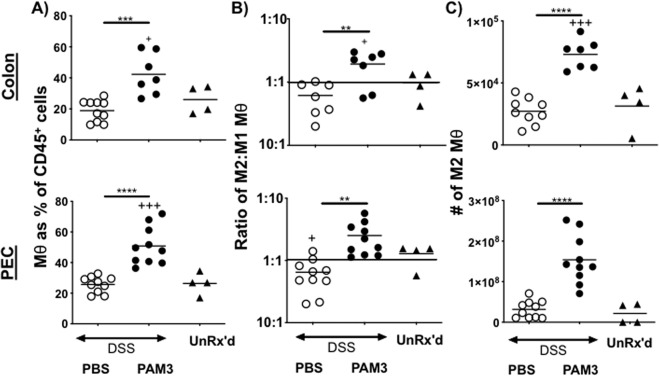
Effect of PAM3 treatment on M2 macrophage frequency. (**A**) Macrophages were identified based on their expression of F480 and shown as a fraction of CD45^+^ cells. (**B**) Ratio of M2:M1 Mθ was determined based on the ratio of CD206^+^:CD206^−^ F480^+^ macrophage. (**C**) The total number of M2 macrophages (CD206^+^ F480^+^ cells) present in the colon and PEC on day 9 is shown. N = 7–10 mice / group. Untreated controls were included for comparison (N = 4). Data represent the mean + SD. *p < 0.05; **p < 0.01; ***p < 0.001 for PAM3 versus PBS. ^+^p < 0.05; ^++^p < 0.01 for PBS or PAM3 versus untreated.

Previous studies established that PAM3 stimulated normal murine and human monocytes to preferentially mature into M2 macrophages^18,19^. As an excess of M1 macrophages can contribute to the pathogenesis of colitis^15^, the possibility that the protective activity of PAM3 was mediated via an increase in the number of M2 macrophages was examined. Immune cells were collected from the peritoneal cavity (PEC) and colon of animals treated with DSS for 8 days. Macrophages were identified by their expression of the F4/80 surface marker while M2 macrophages were identified by the expression of both F4/80 and the CD206 mannose receptor, the latter having been established as a reliable marker of M2 macrophages^21^. PAM3 caused a significant increase in the frequency of F4/80^+^ macrophages in both the PEC and colon (Fig. 2A). This increase was largely due to a preferential increase in the number of M2 macrophages (Fig. 2C). Consistent with previous findings, the inflammation induced by DSS caused the frequency of inflammatory M1 macrophages (which were identified as CD206^−^ F480^+^ cells based on previous work)^10,23^ to rise in PBS treated mice. PAM3 reversed that effect, as shown by changes in both the absolute number and ratio of M2:M1 macrophages (Fig. 2B,C).

**Figure 3:**
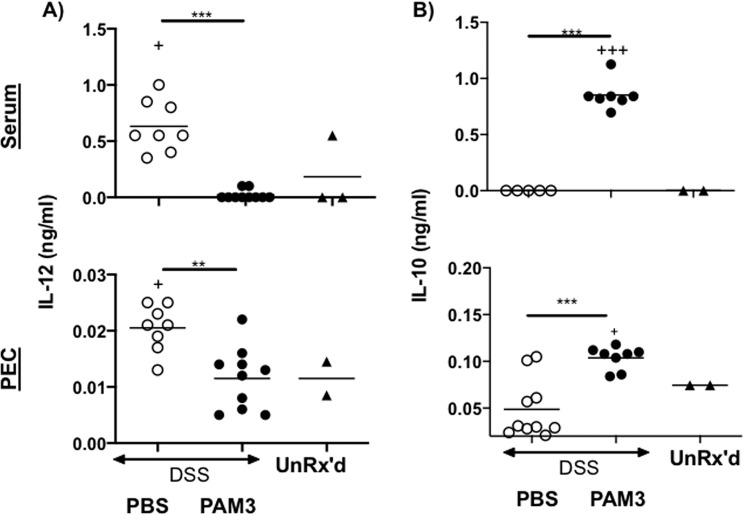
Effect of PAM3 treatment on cytokine production. Serum and PEC were collected on day 9. PEC were cultured *ex vivo* for 24 hr in the absence of further stimulation. Serum and supernatants were analyzed for (**A)** IL-12 and (**B**) IL-10 by ELISA. N = 10 mice/group. Data represent the mean + SD. *p < 0.05; **p < 0.01; ***p < 0.001 for PAM3 versus PBS. ^+^p < 0.05; ^++^p < 0.01 for PBS or PAM3 versus untreated.

Another manifestation of DSS-Induced colitis is an increase in the production of pro-inflammatory cytokines such as IL-12^24^. Serum IL-12 concentrations rose significantly in DSS treated mice (Fig. 3A and p = 0.04). That effect was reversed by PAM3 therapy (p = 0.0002). *Ex vitro* culture of PEC cells confirmed that DSS stimulated the production of IL-12, a process reversed by treatment with PAM3 (Fig. 3A). In contrast, serum levels of the immuno-suppressive cytokine IL-10 were undetectably low in DSS treated mice but rose significantly in recipients of PAM3 (Fig. 3B). Consistent with previous reports showing that M2 macrophages are a major source of IL-10 ^11^, PEC from PAM3 treated mice produced large amounts of that cytokine when cultured *ex vivo* in the absence further stimulation.

### PAM3 generated M2 macrophages protect mice from DSS induced colitis

**Figure 4:**
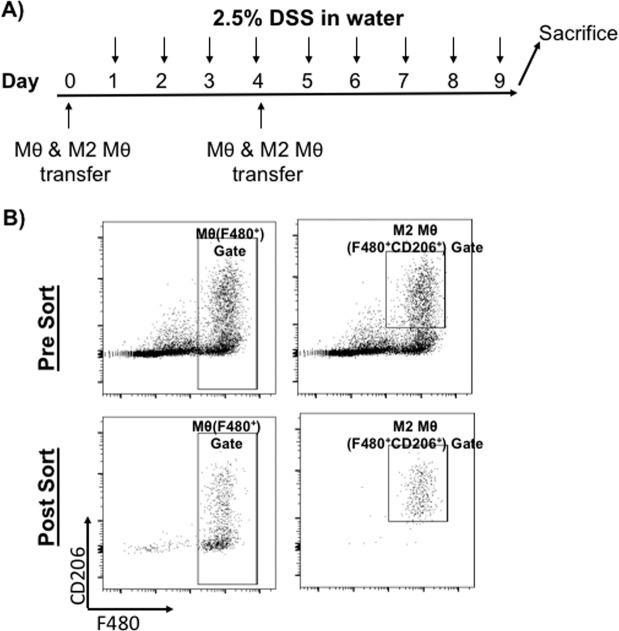
Transfer of PAM3-generated M2 macrophages into DSS treated mice. (**A**) Macrophages were isolated from the PEC of PAM3-treated untreated control mice. On days 0 and 4, 10^6^ total (F480^+^) or 5 × 10^4^ M2 (CD206, F480 double positive) macrophages were transferred into syngeneic recipients who were treated with 2.5% DSS on days 1–9. Animals were sacrificed on day 9. (**B**) Representative flow plots showing the purity of total (F480^+^) and M2 (CD206^+^, F480^+^) macrophage populations pre- and post- FACS sorting.

The studies described above show that PAM3 both boosts the production of M2 macrophages and reduces the severity of DSS induced colitis. To establish that these two outcomes were mechanistically linked, untreated control mice were treated with PAM3 and the M2 macrophages generated were then transferred to DSS treated mice. Preliminary studies established than an optimal yield of M2 macrophages was obtained by treating mice with 2 doses of PAM3 and collecting PEC 1 day later (Fig. 4A and data not shown).

**Figure 5:**
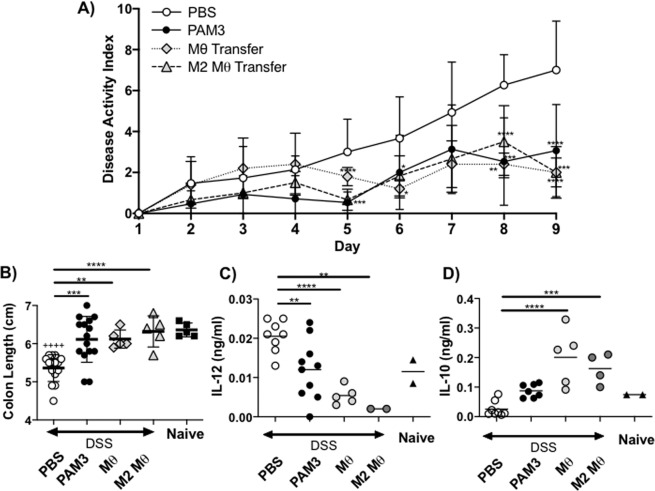
Effect of transferring PAM3-generated M2 macrophages into DSS treated mice. Macrophages were isolated and transferred to DSS treated recipients as described in Fig. 4. (**A**) Recipients were monitored daily for disease progression based on DAI. On day 9, disease severity was assessed by examining colon length (**B**) and by culturing PEC *ex vivo* for 24 hr and analyzing IL-12 (**C**) and IL-10 (**D**) levels by ELISA. N = 10–15 mice/group for PBS and PAM3 treated groups and 5 mice/group for Mac transfer groups. Untreated controls were included for comparison. Data represent the mean + SD. *p < 0.05; **p < 0.01; ***p < 0.001 for PAM3 and macrophage transfer groups versus PBS. ^+ + + +^ p < 0.0001 for PBS versus untreated.

10^6^ total macrophages or 5 ×10^4^ purified M2 macrophages were transferred into recipient mice. The recipients were then treated for 8 days with DSS (Fig. 4A). As in earlier experiments, mice given DSS but nothing else developed severe colitis while those treated with PAM3 developed significantly less severe disease (Fig. 5, p = 0.003). By comparison, recipients of M2 macrophages transferred from DSS treated donors were well protected from disease. The protective effect of these transferred macrophages was confirmed in studies of colon length: M2 macrophages provided the same level of protection from colitis as did PAM3 treatment.

The pattern of cytokine production was also analyzed in recipient animals. Serum levels of IL-12 fell while serum IL-10 levels rose in mice treated either with PAM3 or M2 macrophages (Fig. 5C,D).

## Discussion

This work establishes that the TLR2/1 agonist PAM3 significantly reduces the severity of DSS induced colitis. This beneficial effect of PAM3 arose from its ability to generate M2 macrophages, which when transferred from untreated control donors into DSS treated recipients had the same protective effect as PAM3 alone (Fig. 5). Previous studies established that the frequency of M1 macrophages producing inflammatory cytokines (such as IL-12) is increased in animals and patients with IBD^25,26^. Ample evidence supports the finding that M1 macrophages contribute to the pathogenesis of colitis^6,27^. Although the mechanism(s) responsible for the disease inducing activity of M1 macrophages is not fully understood, recent studies suggest that gram positive commensal bacteria in the gut produce cytokines that recruit proinflammatory macrophages to the colon^28^. Consistent with that possibility, we observed a significant increase in the absolute number and relative frequency of M1 macrophages in both the colon and PEC of mice with DSS colitis (Fig. 2). All animals used in this experiment were born, reared and studied in a single animal room. Microbiome studies of individual mice from reared in that room varied by <1% whether derived from the same or different cages (data not shown).

The literature suggest that M2 macrophages can protect against colitis both by secreting immunosuppressive factors (such as IL-10) that promote tissue repair and by driving epithelial cell regeneration and proliferation^6,27^. Based on the known contribution of macrophages to intestinal homeostasis, macrophage therapy has been proposed as a means of treating IBD. In murine studies, the transfer of *in vitro* generated bone marrow derived M2 macrophages was shown to reduce disease severity^15,29^. Unfortunately, the isolation and transfer of M2 macrophages from the peritoneal cavity of patients is neither practical nor cost efficient. As an alternative, this work investigated whether PAM3 could be used to promote the generation of M2 macrophages as a prophylactic treatment of colitis. PAM3 induces murine monocytes to mature into M2 macrophages over four days *in vivo* {data not shown, section 4.2}. Of note, BMDM cultured with PAM3 for four days *in vitro* primarily generate M1 macrophages^30-32^.This differs from the effect of PAM3 on peripheral blood human monocytes *ex vivo* and murine monocytes *in vivo*, where this TLR agonist preferentially generates M2 rather than M1 macrophages^10,18,19^. Other TLR 2/1 agonists also induce the generation of immunosuppressive macrophages whereas ligands that interact with TLR2/6 heterodimers (such as FSL-1) do not (data not shown). As DSS induces inflammation of the colon immediately upon administration, PAM3 treatment was iniated 1 day earlier, prior to the administration of DSS. By comparison, colitis in patients is characterized by a relapsing/remitting course with much slower kinetics, allowing PAM3 to be used early in the disease process.

Results confirmed the finding that PAM3 stimulates a significant increase M2 macrophage frequency, an effect found in both the colon and PEC (Fig. 2, p < 0.0001 for both). The effect of PAM3 on other immune cells in the colon (such as T and B cells) was also examined yet their frequencies were unchanged (data not shown). This was accompanied by a shift in the M1:M2 ratio when compared to both untreated control animals and mice with untreated colitis (Fig. 2B, p = 0.0072 and 0.0019 for colon and PEC, respectively). PAM3 treatment led to a significant reduction in colon injury, inflammation and the production of proinflammatory cytokines (Figs. 1 and 3). Peritoneal macrophages have been shown to migrate to sites of inflammation^33^. Hence, current findings are consistent with the hypothesis that i.p. administration of PAM3 induces local monocytes to differentiate into M2 macrophages that subsequently migrate to the colon. This would account for the increased number of M2 macrophages in both the peritoneal cavity and colon (Fig. 2C). To establish that M2 macrophages played a key role in PAM3-induced protection, a cell transfer experiment was conducted. To avoid the possibility that contamination by other cell types (or regulatory factors) might be involved, these studies were performed using FACS sorted M2 macrophages from untreated control mice that had never been exposed to DSS. Preliminary experiments identified the dose and frequency of PAM3 needed to optimize the generation of M2 macrophage in these animals (see section 4.2). As a positive control, 10^6^ total peritoneal macrophages were isolated and transferred into DSS treated mice. Consistent with the report of Liu *et al*., that number of macrophages was needed to reduced the severity of murine colitis (^34^ and data not shown). Of importance, equivalent disease control was achieved by transferring only 5 ×10^4^ FACS purified M2 macrophages (Fig. 4A). The transfer of this small number of cells also reduced DAI scores and increased colon length as effectively as 20-fold more total macrophages (Fig. 5A,B). Analysis of M2 macrophages in the colon of recipient mice verified that the transferred cells reached their target organ (data not shown).

M2 macrophages secrete immunosuppressive factors that can protect against colitis^6,14–16^. IL-10 is one such factor: an immunoregulatory cytokine secreted primarily by M2 macrophages^35,36^. Increased levels of IL-10 were present in the serum of mice treated with PAM3 (and recipients of M2 macrophages) and IL-10 was produced by peritoneal cells from these animals during *ex vivo* culture (Figs. 3B and 5D). The importance of IL-10 in maintaining intestinal homeostasis is well documented, with IL-10 KO mice spontaneously developing colitis^6,37^. Similarly, patients with IL-10 receptor deficiency have an enhanced susceptibility to severe IBD^38^.

In addition to increasing IL-10 levels, changes in the M1:M2 macrophage ratio induced by PAM3 caused levels of IL-12 (a proinflammatory cytokine produced by M1 macrophages) to decrease (Figs. 3A and 5C). IL-12 is over- produced by macrophages in the gastric mucosa and has been shown to contribute to the development of IBD^25,26^. IL-12 also supports the production of IFNg which contributes to the initiation of colitis^39^. Neutralizing IL-12 reduces intestinal inflammation^40^ and inhibitors of IL-12 are being evaluated in Phase IIb and III clinical trials for the treatment of IBD^41–43^.

Studies from this and other labs established that CD206/F480 double positive macrophages are functionally M2 as they are phagocytic and produce IL-10 but not IL-12. Those findings are consistent with the elevated levels of IL-10 and decreased levels of IL-12 observed when PAM3 was administration to lupus-prone mice *in vivo* (Fig. 3)^10^. While other mechanisms are possible^44^, findings in this report establish that the ability of PAM3 to protect against colitis can be attributed to its ability to support the generation of M2 macrophages. Transferring purified M2 macrophages from PAM3-treated donors to mice with DSS induced colitis significantly reduced disease severity. This was accompanied by an increase in IL-10 production and reduction in IL-12 production as found in mice treated with PAM3. These findings support the testing of PAM3 for the treatment of colitis.

## Materials and Methods

### Study approval

All rodent experiments were reviewed and approved by the Animal Care and Use Committee of the National Cancer Institute (NCI)-Frederick. All animal experiments were carried out according to these approved protocols.

### Acute colitis model

8 week old female C57/B6Ncr mice (Jackson Laboratories) received 2.5% DSS (molecular mass 36,000–50,000 kDa; Gojira Fine Chemicals) in drinking water for 8 days starting on day 1. Mice were injected every 2 days with 100 μg PAM3 (Invivogen) i.p. starting on day 0 and sacrificed on day 9.

To conduct the macrophage transfer experiments, peritoneal cells were collected from PAM3 treated naïve mice on day 4, after 2 injections of PAM3 with 48 hr apart. These cells were labeled with fluorochrome-conjugated Ab specific for mouse F480 and CD206 (Biolegend). M2 Macrophages were identified by their co-expression of F480 and CD206. Total and M2 Macrophages were isolated using an LSR SORP (BD Biosciences). The purity of the populations was 96 ± 2.6% and 91 ± 2.4% for total and M2 macrophages respectively as determined by post sort FACS analysis. 10^6^ total macrophages and 5 ×10^4^ M2 macrophages were injected i.p. in 100 μl 5% FBS-PBS on days 0 and 4 into mice treated with 2.5% DSS from days 1–9. These animals were sacrificed on day 9.

### Assessment of disease activity and colon length

Disease activity was evaluated using a previously described protocol^7^. Briefly, mice were monitored daily for weight loss, stool consistency and rectal bleeding. These parameters were used to determine the disease activity index (modified from Cooper *et al*.^8^)) which is the sum of the following 3 parameters: weight loss: 1–5% = 1, 5–10% = 2, 10–15% = 3, > 15% = 4; stool consistency: normal stool = 0, loose stool = 2, watery diarrhea = 4; rectal bleeding (assessed by Hemoccult SENSA): negative = 0, faint blue = 1, strong blue = 2, visible blood on feces = 3, gross blood around anus = 4. Colon lengths were measured on day 9 from the ileocecal junction to the rectum.

### Isolation of cells from the lamina propria of the colon

Colons were opened longitudinally and washed gently with PBS. Tissue was cut into small pieces and incubated at 37 °C in RPMI 1640 (Lonza) supplemented with 5% heat-inactivated fetal calf serum (FCS), 100 U/ml penicillin, 100 mg/ml streptomycin, 25 nmol/L HEPES, 1 mmol/L sodium pyruvate, NEAAs, 0.0035% 2-ME and 0.05 mM EDTA (to remove epithelial cells). After three 20 min incubations, tissue was transferred to RPMI 1640 supplemented with 10% FCS, 1.75 mg/ml Collagenase IV (Gibco) and 0.05 mg/ml DNAse I (Roche) and incubated at 37 °C for 1 hr. The cells released by this treatment were isolated by passage through a strainer, centrifugation, and then washed and resuspended in 2% BSA-PBS.

### Flow Cytometry

Single cell suspensions prepared from peritoneal cells and colon were incubated with fluorochrome conjugated Abs specific for murine CD45, F480, CD206 and MHCII for 20 min in 2% BSA-PBS on ice after blocking with Fc-block (Biolegend) for 15 min and then stained^10^. Cells were washed, re-suspended in 2% BSA-PBS and analyzed using LSR Fortessa (BD Biosciences). As previously documented, M2 macrophages were identified based on their co-expression of F480 and CD206 whereas M1 macrophages did not express CD206^10,23^.

### ELISA

ELISA was performed based on the protocol described previously^10^. Immunol 2HB-microtiter plates were coated with mouse anti IL-10 (R&D Systems) or mouse anti-IL12 (BD Biosciences) followed by blocking with 2% BSA-PBS for 2 hr. Culture supernatants or diluted sera were added after washing and incubated overnight at 4 °C. Secondary Ab labeled with biotin were added to plates, and incubated for 2 hr. Next plates were developed by phosphatase-conjugated streptavidin (AKP) followed by the addition of p-nitrophenyl phosphatase (pNPP) substrate (Southern Biotech). Optical density was measured using a SpectraMax M5 microplate Reader and SoftMax Pro Acquisition and Analysis software (Molecular Devices).

### Statistical analysis

Statistical analyses used either 2-tailed unpaired Student t-tests or Dunnett corrected One-way ANOVA multiple comparison tests (GraphPad Software Inc.).
